# Molecular Cloning, Functional Characterization and Nutritional Regulation of the Putative Elongase Elovl5 in the Orange-Spotted Grouper (*Epinephelus coioides*)

**DOI:** 10.1371/journal.pone.0150544

**Published:** 2016-03-07

**Authors:** Songlin Li, Yuhui Yuan, Tianjiao Wang, Wei Xu, Mingzhu Li, Kangsen Mai, Qinghui Ai

**Affiliations:** 1 Key Laboratory of Aquaculture Nutrition and Feed, Ministry of Agriculture, Ocean University of China, Qingdao 266003, People’s Republic of China; 2 Key Laboratory of Mariculture, Ministry Education of China, Ocean University of China, Qingdao 266003, People’s Republic of China; 3 Laboratory for Marine Fisheries and Aquaculture, Qingdao National Laboratory for Marine Science and Technology, Qingdao, China; Universiti Sains Malaysia, MALAYSIA

## Abstract

The enzymes involved in the biosynthesis of long-chain polyunsaturated fatty acids (LC-PUFAs) are widely studied in fish species, as fish are the main source of n-3 LC-PUFAs for human beings. In the present study, a putative gene for *elovl5*, which encodes a key enzyme involved in LC-PUFA synthesis, was cloned and functionally characterized, and its transcription in response to dietary n-3 LC-PUFA exposure was investigated. Moreover, cell transfection and luciferase assays were used to explore the mechanism underlying the regulation of *elovl5*. The full-length cDNA of *elovl5* was 1242 bp (excluding the polyA tail), including an 885 bp coding region encoding a 295 amino acid protein that possesses all of the characteristic features of *elovl* proteins. Functional characterization of heterologously expressed grouper Elovl5 indicated that it effectively elongates both C18 (18:2n-6, 18:3n-3, 18:3n-6 and 18:4n-3) and C20 (20:4n-6 and C20:5n-3) PUFAs, but not the C22 substrates. The expression of *elovl5* was significantly affected by dietary n-3 LC-PUFA exposure: a high n-3 LC-PUFA level repressed the expression of *elovl5* by slightly down-regulating the expression of sterol regulatory element-binding protein (SREBP)-1 and liver X receptor (LXR) α, which are major regulators of hepatic lipid metabolism. Promoter studies showed that grouper *elovl5* reporter activity was induced by over-expression of LXRα but not SREBP-1. This finding suggests that *elovl5* is a direct target of LXRα, which is involved in the biosynthesis of PUFAs via transcriptional regulation of *elovl5*. These findings may contribute to a further understanding of the mechanism underlying the regulation of LC-PUFA biosynthesis in marine fish species.

## Introduction

N-3 long-chain polyunsaturated fatty acids (LC-PUFAs, C≥20 and double bonds≥3), particularly docosahexaenoic acid (DHA, 22:6n-3) and eicosapentaenoic acid (EPA, 20:5n-3), are essential nutrients for humans that play a variety of important roles in promoting cardiovascular health and immune function [1, 2] and in preventing metabolic disease [3]. Due to the lack of Δ12 and Δ15 desaturases responsible for the production of PUFAs from 18:1n-9, PUFA (C≥18 and double bonds≥2), including LC-PUFAs, cannot be synthesized *de novo* from short chain fatty acids in vertebrates [4]. Fish species, especially marine fish, are a primary source of n-3 LC-PUFAs for human beings. However, with increasing use of vegetable oils in aqua feed, such as soybean oil (mainly 18:2n-6), linseed oil (mainly 18:3n-3) and rapeseed oil (mainly18:1n-9), the contents of n-3 LC-PUFAs in farmed fish are significantly decreased due to the lack of LC-PUFAs in vegetable oils [5, 6]. Therefore, the molecular mechanisms regulating the endogenous synthesis of LC-PUFAs in fish species have become an important research topic.

The accepted LC-PUFA biosynthetic pathway in vertebrates involves consecutive desaturation and elongation reactions that convert linolenic acid (18:3n-3) and linoleic acid (18:2n-6) to LC-PUFAs; these reactions are catalyzed by the enzymes fatty acyl desaturase (Fad) and elongation of very long-chain fatty acids (Elovl) [7, 8]. Briefly, LC-PUFAs could be biosynthesized through the classical “Δ6 desaturation—Elovl5—Δ5 desaturation” pathway. Although Δ6 desaturase (Fads2), which catalyzes the first desaturation step in LC-PUFA synthesis, has been widely studied as the rate-limiting enzyme in the LC-PUFA biosynthetic pathway [9, 10], an important role for Elovl5 has also been demonstrated in turbot, *Scophthalmus maximus* [11], and cod, *Gadus morhua* [12]. Given that limited elongation of C18 to C20 PUFAs, rather than limited Δ5 desaturation, accounts for the limited rate of conversion of 18:3n–3 to EPA in a turbot cell line, relatively low Elovl5 activity may be implicated in the cell’s poor ability to synthesize n-3LC-PUFA. Moreover, upon transformation with *elovl5a* from masu salmon, the ability of transgenic zebrafish to biosynthesize LC-PUFAs is elevated [13]. Recently, it has been found that Δ6 Fad in teleosts displays Δ8 activity. The discovery of Δ8 desaturation may indicate the existence of a possible alternative pathway, “Elovl5—Δ8 desaturation—Δ5 desaturation” (the Δ8 desaturation pathway) [14, 15].

*Elovl5* has been successfully cloned and functionally characterized in a variety of teleost fish species, including freshwater fish, marine fish and salmon [12, 16–26]. Those results showed that Elovl5 in fish efficiently elongates C18 (18:4n-3 and 18:3n-6) and C20 (20:5n-3 and 20:4n-6) PUFAs but displays a limited ability to elongate C22 (22:5n-3 and 22:4n-6) PUFAs. Moreover, in studies of southern Bluefin tuna (*Thunnus maccoyii*), Japanese eel (*Anguilla japonica*) and striped snakehead (*Channa striata*), the elongation of 18:3n-3 and 18:2n-6 was further demonstrated to be a characteristic of Elovl5; this finding confirmed the potentially important role of Elovl5 in the Δ8 desaturation pathway.

Nutritional strategies represent a potential option for increasing the endogenous production or retention of n-3 LC-PUFAs in farmed fish [27, 28]. Additionally, the mechanism by which the genes involved in LC-PUFA biosynthesis are generated could be investigated via nutritional experiments. It has been demonstrated that nutrients, especially dietary fatty acids, regulate the transcription of *elovl5* [26, 29–32]. However, to date, the precise mechanisms by which *elovl5* is regulated by nutrients have rarely been investigated in fish. In mammals, PUFAs and their metabolites regulate several transcription factors, such as the nuclear receptors liver X receptors (LXRs) and sterol regulatory element-binding proteins (SREBPs), which modulate the transcription of several target genes [33]. LXRs and SREBPs primarily function in hepatic lipid metabolism via the transcriptional regulation of several key genes [34, 35]. Briefly, SREBP-1c directly stimulates lipogenesis by interacting with its corresponding response element in the promoters of its target genes [35]. Alternatively, LXRs regulate lipogenesis via both direct and indirect mechanisms. Specifically, LXRs directly transcriptionally activate lipogenesis-related genes or indirectly stimulate the expression of lipogenesis-related genes by regulating the expression of *srebp-1c* and certain other transcription factors [36, 37]. In a study of mammals, Qin *et al*. [33] found that mouse *elovl5* was regulated by SREBP-1c and that LXRα indirectly elevated the expression of *elovl5* by regulating SREBP-1c. However, in a study of teleosts, Minghetti *et al*. [38] found that salmon *elovl5* displayed a similar expression profile to that of *LXRα*; their result suggested that salmon *elovl5* is a direct target gene of LXRα. Thus, a greater understanding of the molecular mechanisms regulating the genes involved in LC-PUFA biosynthesis in fish may be useful for elevating endogenous LC-PUFA synthesis.

The orange-spotted grouper, *Epinephelus coioides*, has been widely cultured in Southeast Asia; and this species is a good candidate for intensive aquaculture due to its fast growth performance and huge potential market value [39]. However, to our knowledge, little information is available regarding the mechanism regulating the genes involved in LC-PUFA biosynthesis in this fish species. Only Fads2 has been investigated for its functional characteristics and its expression in response to n-3 LC-PUFA exposure [40]. Thus, a 4-week feeding trial was conducted on grouper larvae to investigate the influence of dietary n-3 LC-PUFA supplementation on the transcription of grouper *elovl5*. Additionally, the grouper elovl5 gene and promoter were cloned and its transcriptional regulatory activity was investigated for the first time. Cell transfection and dual-luciferase reporter assays were also performed in the present study to elucidate the molecular mechanism by which grouper *elovl5* is regulated. These results may contribute to a better understanding the potential regulatory mechanisms in the orange-spotted grouper and may be useful for enhancing endogenous LC-PUFA production.

## Experimental Procedures

### Cloning and sequencing of grouper *elovl5* cDNA

Total RNA was extracted from the isolated grouper liver using Trizol reagent (Takara, Tokyo, Japan). Thereafter, RNA quality was measured as described by Li *et al*. [40]. First-strand cDNA used to synthesize a fragment of *elovl5* using the PrimeScript^™^ RT reagent Kit (Takara, Japan) according to the manufacturer’s instructions. Two degenerate primers (Table 1) were designed to match the highly conserved regions of *elovl5* from other fish (large yellow croaker, cobia and gilthead seabream) to clone a fragment within the coding region via PCR. The PCR protocol was performed in an Eppendorf Mastercycler Gradient thermal cycler (Eppendorf, Hamburg) using the following cycling conditions: 2 min at 94°C, 35 cycles at 94°C for 30 s, 30 s at 54°C, 40 s at 72°C, and a final extension step at 72°C for 10 min. The amplification products were separated, ligated to a vector and sequenced according to the procedures described by Li *et al*. [41]. The cloned nucleotide sequence was then used in a searched of GenBank to confirm its high homology with other *elovl5* cDNAs. After obtaining the partial *elovl5* cDNA sequence, specific primers were designed to obtain the full-length *elovl5* cDNA sequence (Table 1). Subsequently, rapid amplification of cDNA ends (RACE) PCR was performed based on the cDNA reverse transcribed using the SMARTer^™^ RACE cDNA Amplification Kit (Clontech, CA, USA). Briefly, four gene-specific primers, namely Elovl5-F1, Elovl5-F2, Elovl5-R1 and Elovl5-R2 (Table 1), were designed on the basis of the obtained putative *elovl5* cDNA fragment. The full-length cDNA sequence was obtained through two rounds of PCR. The gene-specific primers, Elovl5-F2 and Elovl5-R1, and Universal Primer A Mix (provided in the kit) were used in the first round PCR for 3’ and 5’ RACE. The program for the first-round PCR was as follows: 2 min at 94°C, 30 cycles at 94°C for 30 s, 30 s at 62°C, 1min at 72°C, and a final extension step at 72°C for 10 min. Products from the first-round PCR were used as the template for nested PCR in another 30-cycle reaction under the same thermal conditions as mentioned above. The RACE PCR products were purified, ligated to a vector, and sequenced as described above.

**Table 1:** Sequences of the PCR primers used in this study.

Primer	Sequences (5’-3’)	Purpose
Elovl5-F	GACAACTACCCWCCAACCT	RT primer
Elovl5-R	TCTTCCACCAAAGGTACGG	RT primer
Elovl5-F1	CCCCATCCACACGATTAGAAGGTA	5’RACE primer(inner)
Elovl5-F2	GCGTGTCCTGGCAGTAGAAGTTGTA	5’RACE primer(outer)
Elovl5-R1	TGGACACCTTCTTCTTCATACTACGA	3’RACE primer (inner)
Elovl5-R2	TACAACTTCTACTGCCAGGACACGC	3’RACE primer (outer)
E5-hindIII-F	CCCAAGCTTATGGAGACCTTCAATCATAA	Functional characterization
E5-XhoI-R	CCGCTCGAGTCAATCCACCCTCAGCTTCT	Functional characterization
Elo5-GSP1	CAGGAGTACGGCTGTCTGTGTTTCAT	Cloning of promoter
Elo5-GSP2	GTTTATGATTGAAGGTCTCCATTTGTC	Cloning of promoter
Elo5-XhoI-F	CCGCTCGAGACTATAGGGCACGCGTGGTC	Construction of reporter plasmid
Elo5-HindIII-R	CCCAAGCTTTTGTCACCTGGAAACAGAAAG	Construction of reporter plasmid
SRE-EcorI-F	CCGGAATTCATGAATAGCCTGTCTTTTG	Construction of expression plasmid
SRE-XhoI-R	CCGCTCGAGCTAGCTGTTGGTGACCGT	Construction of expression plasmid
LXR-EcorI-F	CCGGAATTCATGTCCACGCTGTCTGTGAC	Construction of expression plasmid
LXR-XhoI-R	CCGCTCGAGTCACTCGTTGACATCCCAG	Construction of expression plasmid
Elovl5-qF	CAGCTTCGTCCACGTCGTTA	RT-qPCR
Elovl5-qR	CATATGACCGCGCACATCGT	RT-qPCR
SREBP-qF	TGTATCCAACTGTTGAGCACCTG	RT-qPCR
SREBP-qR	CTGTGGCAGTGTGGTCCTAG	RT-qPCR
LXR-qF	TCATGTCAGTCCAGGAGATTGTG	RT-qPCR
LXR-qR	GGTTGTACCGCCGTGATGTC	RT-qPCR
βactin-F	TACGAGCTGCCTGACGGACA	RT-qPCR
βactin-R	GGCTGTGATCTCCTTCTGCA	RT-qPCR

### Sequence and phylogenetic analysis of the putative grouper *elovl5*

The deduced amino acid (AA) sequence of the newly cloned grouper *elovl5* cDNA was aligned with the human (*Homo sapiens*, BC067123), mouse (*Mus musculus*, NM_134255), rat (*Rattus norvegicus*, NM_134382), zebrafish (*Danio rerio*, NM_200453), cobia (*Rachycentron canadum*, FJ440239), and Atlantic salmon (*Salmo salar*, NM_001123567 and NM_001136552) orthologs. Multiple sequence alignment was performed using Mega 4.0. Phylogenetic trees were constructed according to the AA sequences of grouper and vertebrate Elovls using the neighbor joining method [42]. Confidence in the resulting phylogenetic tree branch topology was measured by bootstrapping the data through 1000 iterations.

### Functional characterization of grouper Elovl5 in *Saccharomyces cerevisiae*

The primers Elo5-XhoI-F and Elo5-HindIII-R, containing restriction sites for Xho I and Hind III, were designed and used to obtain the open reading frame (ORF) of the putative grouper *elovl5* gene (underlined in Table 1). The resulting plasmid construct (pYES2-Elo5) was then generated according to a previously described procedure [16, 40]. Next, the purified recombinant plasmid was transformed into *S*. *cerevisiae* competent cells using the S.c. EasyComp Transformation Kit (Invitrogen, UK). Transformation, selection of yeast harboring the transformed plasmids, and yeast culture were performed according to previously described procedures [16, 40, 43]. A single colony of transgenic yeast was grown in *S*. *cerevisiae* minimal medium without uracil supplemented with one of the following substrate fatty acids: 18:3n-3 (0.5 mM), 18:2n-6(0.5 mM), 18:4n-3 (0.5 mM), 18:3n-6 (0.5 mM), 20:5n-3(0.75 mM), 20:4n-6 (0.75 mM), 22:5n-3 (1.0 mM) or 22:4n-6 (1.0 mM). The final concentrations of the substrate fatty acids were identical to those described by Monroig *et al*. [44]. After 2 days in culture, the yeast were harvested, washed and freeze-dried for further analyses. Yeast transformed with pYES2 containing no insert were grown under the same conditions as a control.

### Fatty acid analysis of cultured yeast

Fatty acid methyl ester (FAME) was prepared according to a method that was previously described in detail [43, 45]. FAMEs were separated and quantified using an Agilent HP6890 gas chromatograph (GC) based on the method described by Metcalfe *et al*. [46] with certain modifications [47]. The fatty acids were then identified using a GCMS—QP2010 Ultra (Shimadzu, Japan) as described previously [16, 48]. The proportion of substrate fatty acids that were converted to elongated fatty acid product(s) was calculated as follows: [areas of first product and longer chain products/(areas of all products with longer chain than substrate + substrate area)] [16].

### Cloning of the *elovl5* promoter

Genomic DNA was extracted from the isolated grouper liver using the SQ Tissue DNA Kit (OMEGA) according to the manufacturer’s instructions, followed by DNA quality measurement as described by Li *et al*. [41]. The genomic DNA was then digested with four restriction enzymes (Dra I, Eco RV, Pvu II and Stu I), purified and ligated to GenomeWalker adaptors according to the instructions in the Universal Genome Walker 2.0 Kit user manual (Clontech). Two reverse primers, Elo5-GSP1 and Elo5-GSP2 (Table 1), were designed based on the cloned grouper *elovl5* cDNA sequence to clone the *elovl5* promoter. Nested PCR (two rounds) was performed to isolate the *elovl5* promoter from the genomic DNA using the Universal Genome Walker 2.0 Kit (Clontech). Ap1 and Ap2, which are two additional nested PCR primers that are provided in the kit, were used in the primary and secondary rounds of PCR, respectively. For *elovl5* promoter cloning, the conditions for the primary round of PCR were as follows: 7 cycles of 25 s at 94°C and 3 min at 72°C, 32 cycles of 25 s at 94°C and 3 min at 65°C, and an additional 7 min extension step at 67°C after the final cycle. The conditions for the secondary round of PCR were as follows: 5 cycles of 25 s at 94°C and 3 min at 72°C, 20 cycles of 25 s at 94°C and 3 min at 65°C, and an additional 7 min extension step at 67°C after the final cycle. The PCR products were purified, cloned into a vector, and sequenced as described above.

### Expression and reporter plasmids

PCR fragments corresponding to an NH_2_-terminal segment of 460 amino acids for grouper *srebp-1* (Genbank ID: KU179485) and the ORF of *lxrα* (Genbank ID: KU179483) were amplified using primers containing restriction sites for EcoR I and Xho I, respectively (underlined in Table 1). The amplified DNA was then digested and ligated to a similarly restricted PCS2^+^ vector (Invitrogen, UK) to yield expression plasmid constructs (PCS2-SREBP1 and PCS2-LXRα). The reporter plasmid Elovl5-Luc was constructed by ligating the grouper *elovl5* promoter sequence to the pGL3-basic vector (Promega), and the PRL-CMV Renilla luciferase plasmid (Promega, USA) was used as an internal control. Plasmids for transfection were obtained using the TransGen Plasmid Mini Kit (Beijing, China).

### Cell culture, transfection and luciferase assays

HEK 293T cells were cultured in Dulbecco’s Modified Eagle’s Medium (DMEM; Gibco, USA) supplemented with 10% fetal bovine serum (FBS; Invitrogen) at 37°C in a humidified incubator containing 5% CO_2_. For DNA transfection, cells were seeded in 24-well plates and transfected upon 90–100% confluence. The transfection was conducted using Lipofectamine^™^ 2000 Reagent (Invitrogen, USA) according to the manufacturer’s instructions. Briefly, the plasmid mixture (expression plasmid, 0.3 μg, reporter gene plasmid, 0.1 μg; and PRL-CMV Renilla luciferase plasmid, 0.01 μg) and Lipofectamine^™^ 2000 (1.0 μL) were co-transfected into the cells. All assays were performed using three independent transfections. Firefly and Renilla luciferase activities were measured using a Dual Luciferase Reporter Assay System (Promega, USA). Briefly, 24 h after transfection, the HEK293T cells were washed twice with PBS (100 μL), followed by lysis with 1× passive lysis buffer (PLB) (100 μL) at room temperature for 10 min. The cell lysate (20 μL) was then transferred to a new tube, followed by the addition of luciferase assay reagent II (50μL) and 1× Stop & Glo Substrate (50 μL) in sequence. Finally, the firefly and Renilla luciferase activities were independently measured using an InfiniTE200 microplate reader (Tecan, Switzerland).

### Animal experiments

Five isoproteic (58% crude protein) and isolipidic (16% crude lipid) diets containing distinct levels of n-3 LC-PUFAs (DHA+EPA) (0.52, 0.94, 1.57, 1.97 or 2.43%) were formulated by supplementation of DHA-enriched oil and EPA-enriched oil to investigate the effects of n-3 LC-PUFAs on the regulation of grouper *elovl5*(S1 and S2 Tables). The diets used in the present study were broken into sizes ranging from 250 to 380 μm for the larvae between 29 and 45 days after hatching (DAH) and 380 to 550 μm for the larvae thereafter. The grouper larvae (70±2 mg, 29 DAH) were obtained from a local rearing farm and were reared in white plastic tanks (water volume, 100 L) at Hainan Virtue Wealth Aquatic Technology Development, located in Yandun, Hainan, China. Triplicate groups of grouper larvae (29 DAH) at a stocking density of 120 individuals per tank were fed to apparent satiation six times daily for 4 weeks. At the end of the feeding trial, the larvae were fasted for 24 h and anesthetized with eugenol (1:10,000) (Shanghai Reagent Corp., Shanghai, China). Afterward, visceral mass from five fish per tank was isolated, pooled into a 1.5 mL RNase-free tube (Axygen, USA), frozen in liquid nitrogen and then stored at -80°C for subsequent gene expression analysis. Additionally, various tissues (eye, brain, heart, liver, kidney, stomach, intestine and muscle) were isolated from nine grouper individuals (10–20 g) to investigate the tissue distribution of *elovl5*. All experiments were performed according to the standard operating procedures (SOPs) in the Guide for the Use of Experimental Animals of Ocean University of China (OUC), and all animal care and use procedures were approved by the Institutional Animal Care and Use Committee of OUC (Permit Number: 20001001).

### Real-time quantitative PCR (RT-qPCR) analysis

The transcription of the putative grouper *elovl5* gene in various tissues (eye, brain, heart, liver, kidney, stomach, spleen, intestine and muscle) and of *elovl5*, *srebp-1* and *lxrα* in visceral mass from grouper larvae fed different diets was measured via RT-qPCR using β-actin (GenBank ID: AY510710) as the reference gene. The stability of β-actin expression was confirmed. Specific primers for RT-qPCR analysis of *elovl5*, *srebp-1*, *lxrα* and *β-actin* (Table 1) were designed using Primer Premier 5.0. Amplification was performed using a quantitative thermal cycler (Mastercycler ep realplex, Eppendorf, Germany) according to a previously described procedure [41]. Standard curves were generated using five different dilutions (in triplicate) of the cDNA samples, and the amplification efficiency was analyzed as follows: E = 10^(–1/Slope)^-1. Because the absolute ΔCt values of the slopes were less than 0.1, the ΔΔCt calculation method could be used for the relative quantification of target genes. The expression levels of the target genes were calculated using the 2^–ΔΔCt^ method described by Livak and Schmittgen [49].

### Statistical analysis

The results are presented as the means ± standard error of the mean (S.E.M.). All data were subjected to one-way ANOVA and correlation analysis where appropriate using SPSS 19.0 for Windows. Duncan’s multiple range test was chosen as a multiple comparison test, and a significance level of 5% was used.

## Results

### Molecular cloning and phylogenetic analysis of grouper *elovl5*

**Fig 1:**
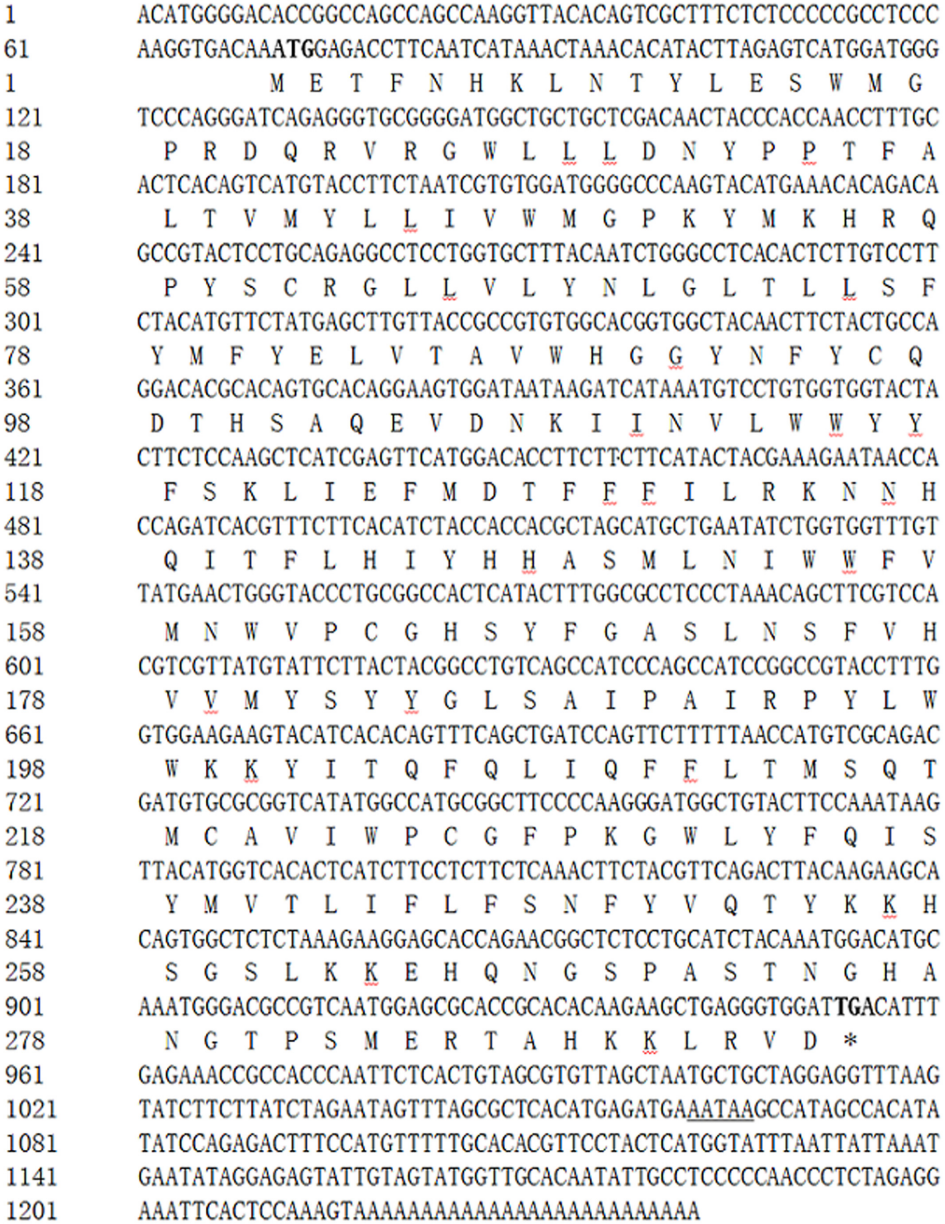
Nucleotide and deduced AA sequences for the *elovl5* gene. Uppercase letters indicate the translated region, and lowercase letters indicate the untranslated region. The start codon (ATG) and the stop codon (TAG) are in bold. Double-underlined letters indicate the polyadenylation signal (AATAA).

The grouper *elovl5* cDNA was 1242 bp in length (excluding the polyA tail) and contained a 70 bp 5’-untranslated region (UTR), an 885 bp coding region encoding a 295 AA protein (GenBank ID: KF006241) and a 287 bp 3’-UTR (Fig 1). BLAST analysis of the deduced AA sequence of grouper Elovl5 indicated that Elovl5 in orange-spotted grouper shares sequence homology with Elovl5 of other teleosts, such as the large yellow croaker (*Larimichthys crocea*, 96%), the cobia (*R*. *canadum*, 95%), the Atlantic salmon (*S*. *salar*, 84%), and the zebrafish (*D*. *rerio*, 77%), as well as greater than 70% identity with Elovl5 of humans (*H*. *sapiens*, 70%), mice (*M*. *musculus*, 71%) and cattle (*Bos taurus*, 71%).

**Fig 2:**
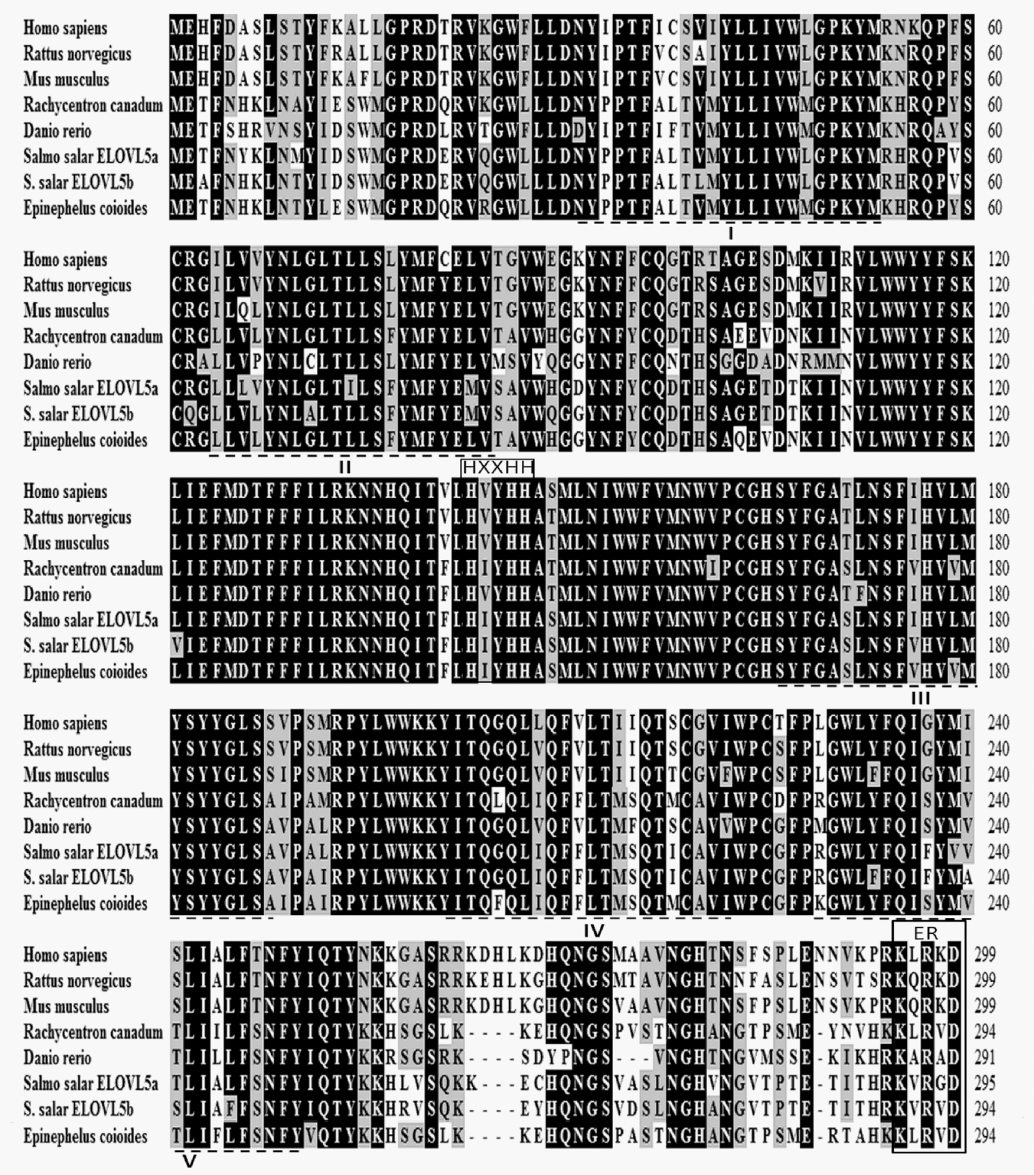
Comparison of the deduced AA sequence of Elovl5 from orange spotted grouper with that from other fish, mice and humans. The AA sequences were aligned using ClustalX, and identity/similarity shading was based on a 75% identity threshold. Identical residues are shaded black, and similar residues are shaded gray. The conserved HXXHH histidine box motif, five (I–V) putative membrane-spanning domains and the ER retrieval signal predicted by Tvrdik *et al*. [50] are also indicated.

**Fig 3:**
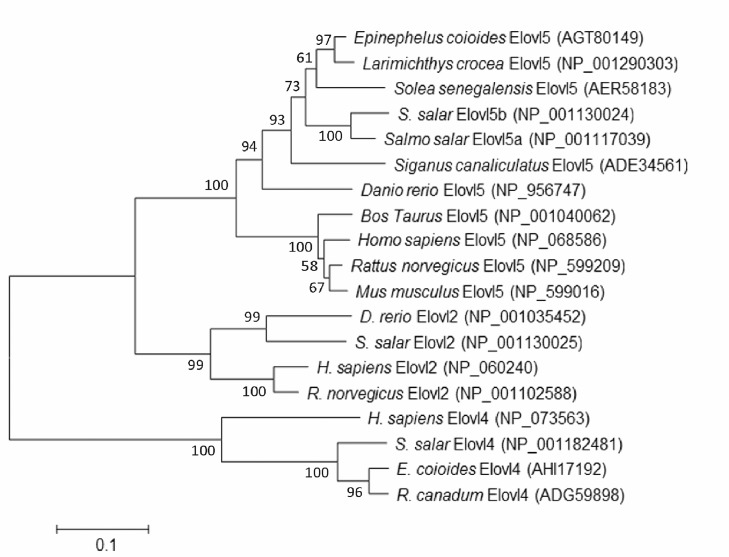
Phylogenetic tree comparing grouper Elovl5 with elongase proteins from other organisms. The phylogenetic tree was constructed using the neighbor joining method [42] in MEGA4. The horizontal branch length is proportional to the AA substitution rate per site. The numbers represent the frequencies (%) with which the tree topology presented was replicated after 1000 iterations.

Characteristically, the proteins deduced from the newly cloned *elovl5* cDNA contained one histidine box (HXXHH) and five membrane-spanning domains possessing single lysine and arginine residues, or KXRXX, at the C-terminus (Fig 2). Phylogenetic analysis clustered grouper *elovl5* with several other elongases of fish and mammals (Fig 3). Furthermore, phylogenetic analysis indicated that grouper *elovl5* clustered closer to *elovl5* of other teleosts than to the *elovl2* and *elovl4* cluster.

### Functional characterization of the putative grouper Elovl5 in *S*. *cerevisiae*

**Fig 4:**
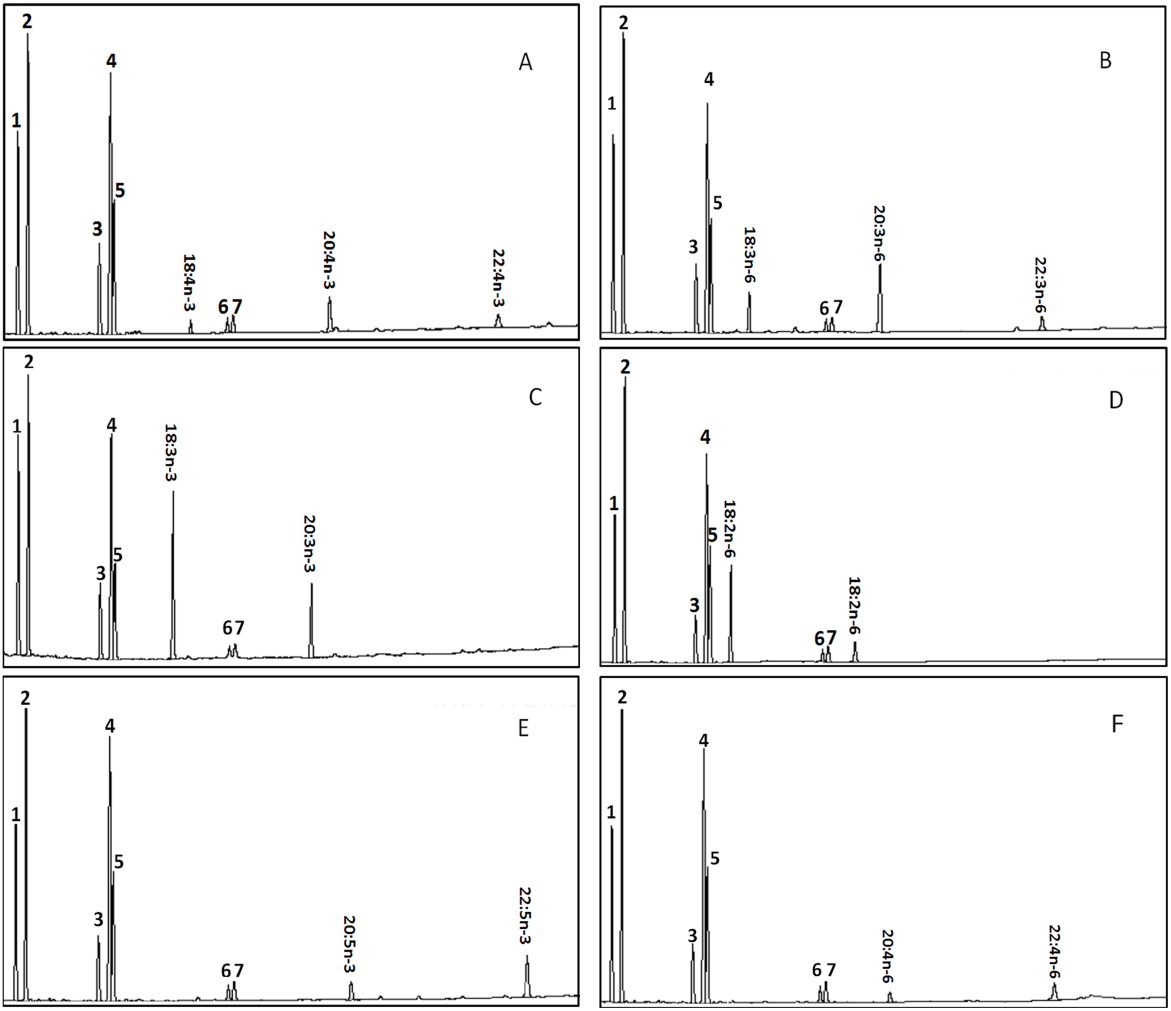
Functional characterization of the putative grouper Elovl5 in transgenic *S*. *cerevisiae* grown in the presence of the substrate fatty acid 18:4n-3 (Panel A), 18:3n-6 (B), 18:3n-3 (C), 18:2n-6 (D), 20:5n-3 (E) or 20:6n-3 (F). Fatty acids were extracted from yeast transformed with the pYES2 vector containing the ORF of the putative Elovl5 as an insert. Peaks 1–4 represent the primary endogenous fatty acids of *S*. *cerevisiae*, specifically 16:0 (1), 16:1n-7 (2), 18:0 (3) and 18:1n-9 (4). The remaining major peaks correspond to the exogenously added fatty acid and the products of its elongation (18:1n-7 (5), 20:1n-9 (6) and 20:1n-7 (7) resulted from the elongation of 16:1n-7, 18:1n-9 and 18:1n-7, respectively). Vertical axis, flame ionization detector (FID) response; horizontal axis, retention time.

The putative Elovl5 protein was functionally characterized by determining the fatty acid profiles of *S*. *cerevisiae* transformed with either an empty pYES2 vector or the resulting plasmid construct (pYES2-Elo5) and grown in the presence of potential substrate fatty acids, including 18:4n-3, 18:3n-6, 18:3n-3, 18:2n-6, 20:5n-3, 20:4n-6, 22:5n-3 and 22:4n-6. The fatty acid composition of the yeast transformed with only pYES2 consisted of the four main fatty acids, specifically 16:0, 16:1n-7, 18:0 and 18:1n-9, together with any exogenously added fatty acid (data not shown) [16, 48]. Grouper Elovl5 displayed the ability to elongate C18 and C20 PUFA substrates, but not the C22 substrates. The conversion rate of 18:4n-3 to 20:4n-3 or 22:4n-3 was 77.76%; a more modest conversion rate (69.25%) was observed for 18:3n-6 (Fig 4; Table 2). Furthermore, relatively high conversion rates were found for 20:5n-3 (75.03%) and 20:4n-6 (66.27%) (Fig 4; Table 2). In addition, relatively weak activity was observed for elongation of 18:3n-3 (36.44%) and 18:2n-6 (15.97%), which may serve as substrates for Δ8 desaturation (Fig 4; Table 2). Moreover, the endogenous mono-unsaturated fatty acids (MUFAs), 16:1n-7, 18:1n-7 and 18:1n-9, could be elongated to 18:1n-7, 20:1n-7 and 20:1n-9, respectively (Fig 4).

**Table 2:** Activity of the putative grouper fatty acyl elongase in yeast. The proportion of substrate fatty acids that were converted to elongated fatty acid product(s) was calculated as follows: [areas of first product and longer-chain products/(areas of all products with longer chain than substrate + substrate area)].

Fatty acid substrate	Product	Conversion (%)	Activity
18:4n-3	20:4n-3	77.76	C18 →20
	22:4n-3	17.35	C20→22
18:3n-6	20:3n-6	69.25	C18 →20
	22:3n-6	14.90	C20→22
20:5n-3	22:5n-3	75.03	C20→22
20:4n-6	22:4n-6	66.27	C20→22
18:3n-3	20:3n-3	36.44	C18 →20
18:2n-6	20:2n-6	15.97	C18 →20

### Tissue expression of the putative *elovl5*

**Fig 5:**
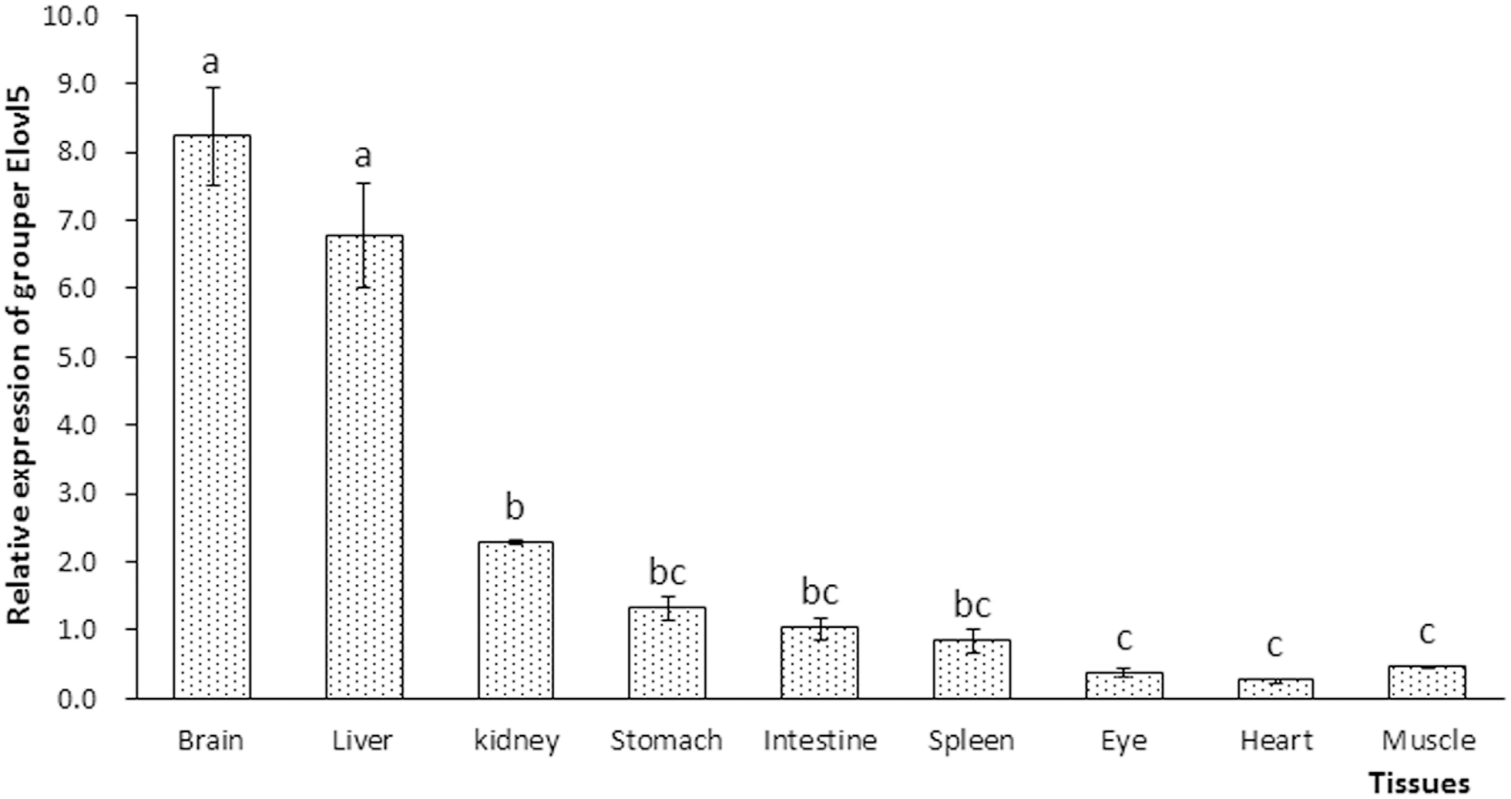
Tissue expression of Elovl5 in the orange-spotted grouper. The results are expressed as the means±standard error (n = 3). Different letters above the bars denote significant (*P*<0.05) differences between tissues.

The tissue distribution of grouper *elovl5* was confirmed via RT-qPCR. The transcription of *elovl5* was detected in eye, brain, heart, liver, kidney, spleen, stomach, intestine and muscle tissue. Relatively high transcription of *elovl5* was observed in brain and liver tissue (Fig 5).

### Expression analysis of *elovl5*, *srebp-1* and *lxrα* in response to n-3 LC-PUFA exposure

**Fig 6:**
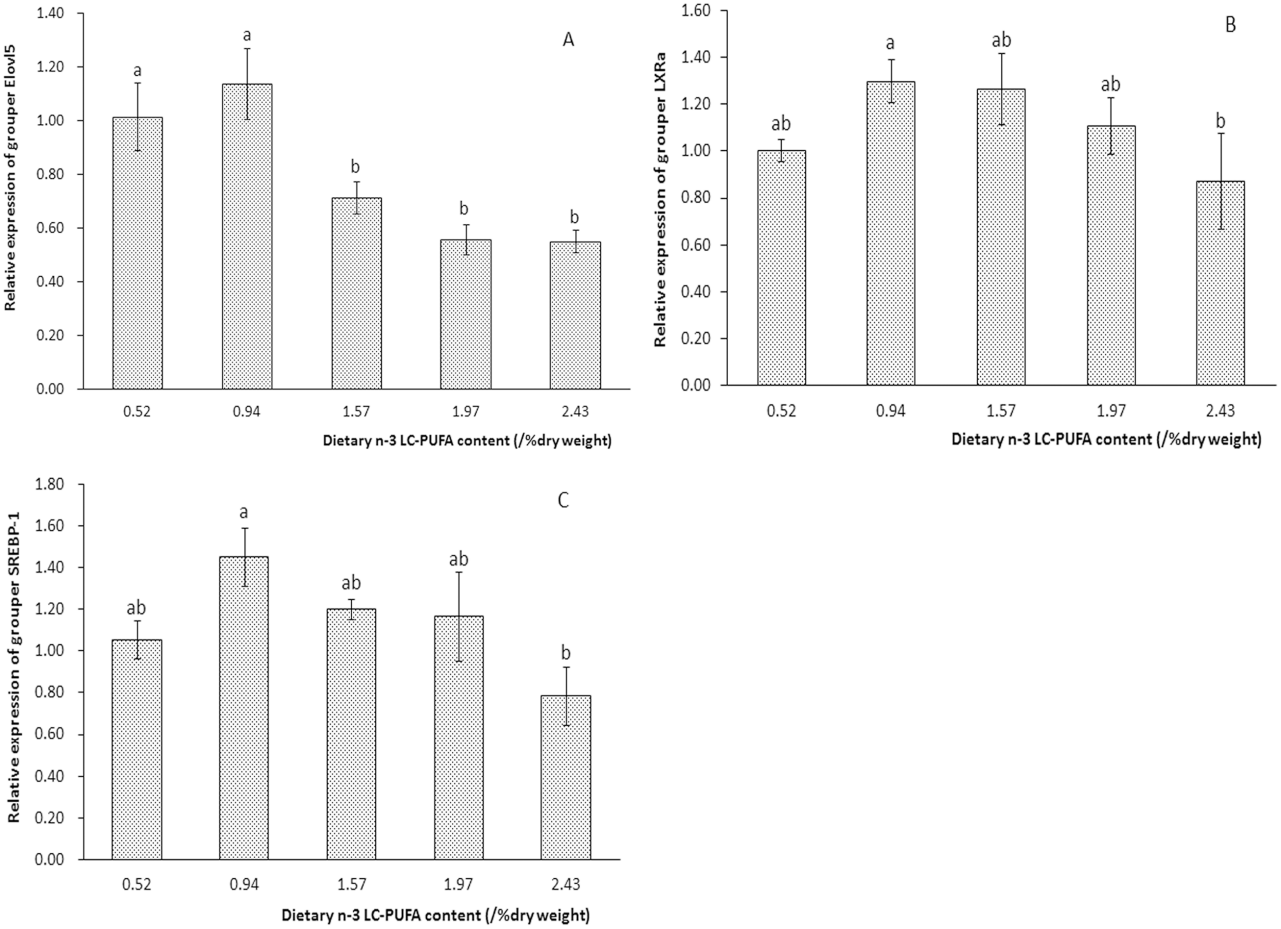
Relative mRNA expression of *elovl5* (A), *lxrα* (B) and *srebp-1* (C) in visceral mass from grouper larvae (29DAH) fed a diet containing various n-3 LC-PUFA concentrations for 4 weeks. Relative mRNA expression was evaluated via RT-qPCR. The values are presented as the means ± S.E.M. (n = 3). Bars for the same gene bearing different letters are significantly different based on Duncan’s test (*P*<0.05).

The relative mRNA expression of *elovl5* in visceral mass from grouper larvae was remarkably affected by dietary n-3 LC-PUFA supplementation (*P*<0.05). The expression of *elovl5* in larvae fed a diet containing 0.94% n-3 LC-PUFAs was comparable to that in larvae fed a diet containing 0.52% n-3 LC-PUFAs; however, *elovl5* expression was significantly higher in these two groups than in the remaining groups (Fig 6a). Additionally, exposure to a high (2.43%) n-3 LC-PUFA content slightly depressed the expression of *srebp-1* and *lxrα* (Fig 6b and 6c).

### Dual-luciferase reporter assays

**Fig 7:**
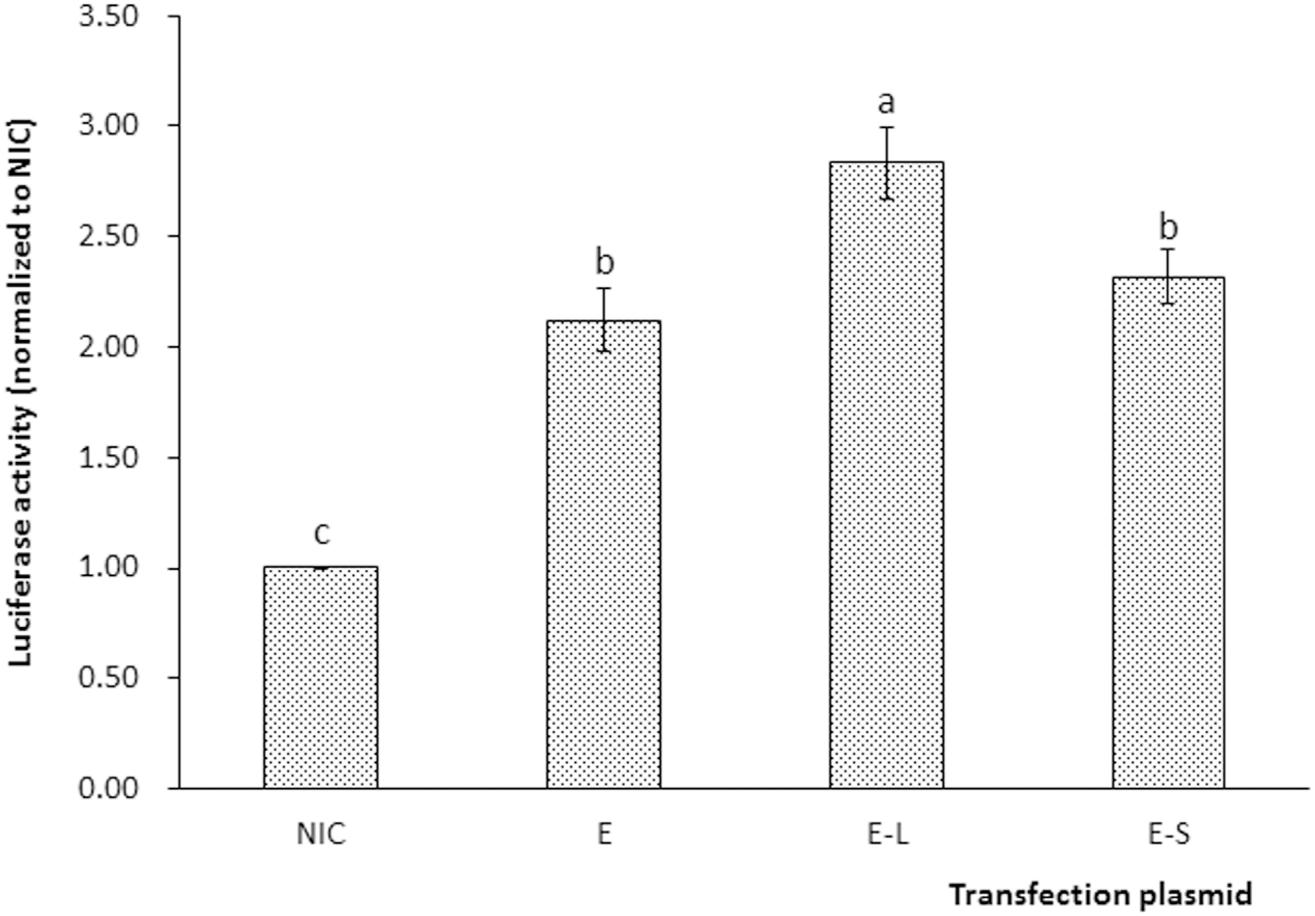
Results of the dual-luciferase reporter assays. The type of transfection plasmid applied to each group was as follows: NIC: PGL3-basic+PCS2+PRL-CMV; E: PGL-Elo5 promoter+PCS2+PRL-CMV; E-L: PGL-Elo5 promoter+PCS2-LXRα+PRL-CMV; and E-S: PGL-Elo5 promoter +PCS2-SREBP-1+PRL-CMV.

To characterize the molecular mechanism involved in the regulation of *elovl5*, the promoter of grouper *elovl5*, a 1890 bp sequence upstream of the initiation codon of *elovl5*, was cloned and deposited in the GenBank promoter sequence database under accession no. KU179484. The results of dual-luciferase reporter assays showed that Elovl5 reporter activity was 2.21-fold higher than the control activity (measured for the empty vector PGL3-basic). Moreover, grouper Elovl5 reporter activity was significantly elevated by over-expression of LXRα, but not SREBP-1 (Fig 7).

## Discussion

The overall objective of the present study was to elucidate the nutritional regulatory mechanisms controlling LC-PUFA synthesis in marine fish. In a previous study, it was found that 1.57% n-3 LC-PUFA could meet the minimum requirement of grouper larvae, which indicates the low endogenous LC-PUFA biosynthetic ability of grouper larvae [40]. Low LC-PUFA biosynthetic ability impacts the health and the flesh quality, and especially the DHA and EPA contents, of this fish species, as has been confirmed in a study by Lin *et al*. [51]; thus, this should also affect the aquaculture industry related to orange-spotted grouper. Low endogenous LC-PUFA biosynthetic ability may be due to the low efficiency and activities of the enzymes involved in the LC-PUFA biosynthetic pathway, and a previous study has demonstrated that the low activity of Δ6 Fad may account for it [40]. In the present study, another vital enzyme, Elovl5, involved in the LC-PUFA biosynthetic pathway was cloned and functionally characterized. The nutritional regulatory mechanism was also explored. This study may contribute to elevate the endogenous LC-PUFA biosynthetic ability and promote the development of aquaculture industry related to orange-spotted grouper.

Elovl5 plays a critical role in the elongation of C18 and C20 PUFAs, and this protein has been considered as a likely key enzyme in the suggested Δ8 desaturation pathway [14]. In the present study, grouper *elovl5* was cloned and functionally characterized for the first time. Moreover, the regulation of *elovl5* by *lxrα* in response to dietary fatty acid supplementation was newly clarified. These studies may provide novel clues regarding the mechanism by which *lxrα* regulates the biosynthesis of PUFAs in marine fish species.

The *elovl5* cDNA isolated from grouper exhibited all characteristic features of Elovl protein family members, including a single histidine box (HXXHH), a C-terminal ER retrieval signal (KXRXX) and five transmembrane regions [52]. Phylogenetic analysis suggested that the deduced AA sequence displays a closer physiological relationship with its corresponding orthologs from other teleosts and mammals than with other Elovl family members in fish species, such as Elovl2 and Elovl4. This result confirmed that the cloned *elovl* cDNA encoded the Elovl5 protein. Furthermore, we determined the specific functions of this *elovl* cDNA via heterologous expression in *S*. *cerevisiae*.

*Elovl5*-encoding cDNAs have been successfully isolated and functionally characterized in many fish species, and all fish Elovl5 proteins display elongation activity toward C18 (18:4n-3 and 18:3n-6) and C20 (20:5n-3 and 20:4n-6) PUFAs as described above. In a study of southern Bluefin tuna, Elovl5 was also demonstrated to exhibit elongation activity toward 18:2n-6 and 18:3n-3 [11], which had not been examined in previous studies. The discovery of Δ8 activity indicated that Elovl5 is a potential key enzyme in the suggested “Δ8 pathway” [14, 15]. Since then, whether 18:3n-3 and 18:2n-6 can be elongated by fish Elovl5 has drawn much attention. In studies of Japanese eel and striped snakehead, the important role of Elovl5 in the potential “Δ8 pathway” was confirmed [25, 26].

The ability of grouper Elovl5 to elongate C18 (18:4n-3 and 18:3n-6) and C20 (20:4n-6 and 20:5n-3) PUFAs confirmed its role in the classical Δ6 desaturation pathway. In addition, the ability of grouper Elovl5 to elongate 18:3n-3 and 18:2n-6 confirmed the existence of an alternative Δ8 desaturation pathway in grouper. Previous studies have found that certain teleost Elovl5 isoforms exhibit little elongation activity toward C22 PUFA substrates [12, 16–26]. Moreover, no activity of grouper Elovl5 toward C22 substrates was found in the present study, and studies of rainbow trout and Japanese eel have reported identical results [17, 25]. This observation suggests that grouper Elovl5 is not involved in the Sprecher pathway for DHA synthesis. Additionally, grouper Elovl5 showed no differential preference between C18 (18:4n-3 and 18:3n-6) and C20 (20:4n-6 and 20:5n-3) PUFAs among the PUFAs examined; these results were similar to the reported results for southern Bluefin tuna, tilapia and turbot [12, 22]. However, Elovl5 isolated from zebrafish, Atlantic salmon, catfish, meagre and striped snakehead exhibit a preference for C18 (18:4n-3 and 18:3n-6) PUFA substrates [12,16] and Elovl5 isolated from cobia, Asian sea bass, northern Bluefin tuna and rabbitfish display a preference for C20 (20:4n-6 and 20:5n-3) PUFA substrates [20,23,24]. However, the underlying reason accounting for these differential preferences remains unclear. In addition, grouper Elovl5 is more likely to elongate n-3 PUFA substrates than n-6 PUFA substrates. This result indicates that vegetable oil enriched in n-3 PUFAs or with a relatively high ratio of n-3 to n-6 PUFAs should be preferentially used to enable the full function of Elovl5. The elongation activities of grouper Elovl5 toward C18 (18:4n-3 and 18:3n-6) and C20 (20:5n-3 and 20:4n-6) PUFAs are relatively high (greater than 60%), so these activities would provide abundant substrates for DHA biosynthesis, regardless of the expression levels of Δ6Fad and Elovl5.

It is well known that marine fish species, in contrast to freshwater species and salmon, exhibit no or little LC-PUFA biosynthetic capacity due to their lack of or low levels of enzymes actively involved in the LC-PUFA biosynthetic pathway [53]. In previous studies of Δ6Fad, it was demonstrated that the lower expression of Fads2 in marine fish species, European sea bass and Atlantic cod, than in salmonids may result from differences binding sites in the Fads2 promoter region [54, 55]; these differences in the binding sites in the *elovl5* promoter between marine fish species and freshwater species may account for the reduced LC-PUFA biosynthetic capacity of marine fish species. Elucidating the differences in promoter sequences between marine and freshwater species would provide further helpful information for enhancing endogenous LC-PUFA biosynthesis.

Marine fish species primarily rely on exogenous intake to meet their daily requirements for LC-PUFAs. However, an enhanced endogenous ability to produce LC-PUFAs would alleviate the dependence of these species on exogenous LC-PUFA intake. Previous studies have found that *elovl5* is regulated by lipid levels and fatty acid profiles [26, 29–32] and that its regulation involves two transcription factors, namely, *srebp-1* and *lxrα* [33, 38]. In the present study, high n-3 LC-PUFA supplementation down-regulated the transcription of *elovl5* significantly. This observation reflects the existence of negative feedback regulation in the LC-PUFA synthetic pathway. A similar result has been found in studies of large yellow croaker [32], common carp [30] and striped snakehead [26]. This feedback suppression of *elovl5* expression may be largely attributable to the alteration of *srebp-1* or *lxrα* transcription in response to n-3 LC-PUFA exposure. In accordance with this assumption, the expression of *srebp-1* and *lxrα* is slightly inhibited by n-3 LC-PUFAs. In mammals, it was reported that n-3 LC-PUFAs, including DHA and EPA, may regulate SREBP-1 at both the transcriptional and the post-translational levels in an LXRα-dependent manner [56]; this phenomenon may account for the similar trends in *srebp-1* and *lxrα* expression in the present study. The mechanism regulating *elovl5* has also been investigated, and it has been demonstrated that mouse *elovl5* is directly regulated by *srebp-1*. However, salmon *elovl5* is probably directly regulated by *lxrα*, and not *srebp-1* [37]. Therefore, dual-luciferase reporter assays were performed in the present study to clarify the mechanism underlying the regulation of grouper *elovl5*. For this purpose, the promoter of grouper *elovl5* together with LXRα and SREBP-1 expression vectors was transfected into HEK293T cells. The increased activity of the *elovl5* reporter compared with the control indicated activation of the grouper *elovl5* promoter by the luciferase reporter genes. Moreover, grouper Elovl5 reporter activity was significantly elevated by over-expression of LXRα, but not SREBP-1. This finding suggested that *elovl5* was positively regulated by *lxrα* via an LXR response element in its promoter and that grouper *elovl5* is not directly regulated by *srebp-1*. These results are consistent with the predicted regulation of *elovl5* in Atlantic salmon [38], in which *elovl5* was proposed to be regulated by *lxrα* at the transcriptional level. Elevating the production of endogenous LC-PUFAs via *lxα* may exert an important negative feedback function in LC-PUFA biosynthesis. The direct stimulatory role of *lxrα* in grouper *elovl5* transcription provides a means for enhancing endogenous LC-PUFA production via not only nutritional strategies but also genetic modification.

In summary, grouper Elovl5 exhibited all characteristic features of Elovl proteins. Functional characterization of grouper Elovl5 via heterologous expression revealed that the enzyme displays elongation activity toward C18 and C20 PUFA substrates, although no activity of grouper Elovl5 toward C22 substrates was detected. The mRNA expression of *elovl5* was significantly decreased by high LC-PUFA levels, and this decrease was likely mediated by *lxrα*.

## Supporting Information

S1 Dataset ORF sequences of grouper *srebp-1* and *lxrα* genes and segment sequence of *elovl5* promoter. (PDF)

S1 Table Formulation and proximate analysis of the experimental diets (% dry weight). (PDF)

S2 Table Fatty acid composition of the experimental diets (% total fatty acids). (PDF)
