# Supplementary material for: Molecular Cloning, Functional Characterization and Nutritional Regulation of the Putative Elongase Elovl5 in the Orange-Spotted Grouper (Epinephelus coioides)
Source: PLoS One. 2016 Mar 7;11(3):e0150544. doi: 10.1371/journal.pone.0150544 (PMC4780818; doi:10.1371/journal.pone.0150544)
Supplement: S1 Dataset — (PDF) [file pone.0150544.s001.pdf]

>grouper LXRα ORF
ATGTCCACGCTGTCTGTGACTGATATCCCAGATGTTGGTCATGATGAGAGTAAGGTGTTTGACGGGGCCTCT
GAGCTGCAGCTGGACTGCATGGTTGAGGAGAGCAGTGGAAGCACCACAATGAAGCACGATGGCCTGCTGT
CGCTGGCTGACCTCTCCCAGCCAGATGACTTCCCCGTCCCCCCCCATAATGGCCCTTCACTAACTGACATGAG
CAGCCCTCTGCCGGTAGAGCCGAGCGACATTAAGTTGGATCCATCTGCAGGTGACACATCTGCCAGCATAGA
TGGCCAGCCAGTGAAGAGAAAGAAGGGGCCCGCGCCAAAGATGCTCGGCAATGAGGTGTGCAGTGTATGT
GGTGACAAGGCCTCTGGTTTCCATTACAACGTGTTGAGCTGTGAGGGCTGCAAGGGCTTCTTTCGGCGCAG
CGTCATTAAAAGTGCCCAGTACAACTGCAAAAACAACGGCCGCTGTGAAATGGACATGTACATGCGGCGCA
AGTGCCAGCAGTGCCGCCTGCGCAAGTGTCGGGAGGCGGGCATGCTGGAGCAGTGTGTGCTCTCTGAGGA
GCAAATCAGACTGAAGAAGATGAAGAAGCAGCAGGAGGAGGAAACAGCCCGCACGTCCACAGTGGTCAC
CCCCACCCCTCCGCAGGAAACAGCCGCGCTCGATCCACAGCAGCAGGAGATGATTGAGAAGCTGGTGGCC
ATGCAGAAGCAATGCAACAAAAGGTCTTTCCTTGACCGACCGAAAGTGACACCGTGGCCACAGAGTCAGG
ACCTGCAGAACCGAGAAGTGCGTCAGCAGCGGTTCGCCCACTTCACTGAGCTAGCAATCATGTCAGTCCAG
GAGATTGTGGATTTTGCTAAGCAGCTTCCTGGTTTCCTGGAGCTCACGAGGGAAGACCAGATCGCTCTACT
GAAGACATCAACCATTGAGATTATGCTGCTTGAGACATCACGGCGGTACAACCCTGCTATCGACAGCATTAC
ATTTCTGAAGGATTTCAGCTACAATAAGGAGGATTTCGCCAAAGCAGGGCTTCAGTTTGAGTTCATTAACCC
CATCTTTGAGTTTTCTAAAGGAATGAACGACCTGCACCTGGATGAGGCAGAATATGCGCTGCTTATTGCCATC
AACATCTTTTCTGCAGATCGTCCGAATGTGCAGGATCACGATCTGGTGGAGAGGCTGCAGCAGCCCTATGTG
GACGCACTGCGCTCTTACATCATGATAAAGAGACCAAATGATCATTTGATGTTTCCCCGTATGCTGATGAAGT
TGGTGAGCCTCCGCACACTAAGCAGCGTCCACTCGGAGCAGGTTTTCGCCCTTCGCCTCCAGGACAAGAAG
CTGCCCCCGCTGCTCTCTGAAATCTGGGATGTCAACGAGTGA
>grouper elovl5 promoter
CTGTTTGAATCCATAACCAAGGACAGACCGATCAAGCAAGCAGTAATGCACCAACATAACTTGACAGCTAAG
GCAAGAGAGCATGCACAAAGATTTAGAAGTGTGTTTTGTCAAAGTAAATTGTAGCAGCGCTGCCTGTGCCA
GACAAGGTTATGCAATTATTAAAAACCTGGAGTACATTAGTGGTAATAAATCTACACATTTCATGACCTTCAAC
AATTTAAAAGCCAGAGAATCACTACACAAACGTCTGGTTATTGTCCGGACGCACCTGCCTGCTGGCTGCTTG
TTTTACAGACACATTTGATCTGTTGTTTGAAACCACAGCGGTGTGGAAGATCAGTTATTGGGGTGTGAGATG
TATTAGAGAGTGTGAGTGGAGAGGCTGGAGCACAGACATTTACAACAGACACAATAGGGCTGGCCCGGTAT
ATCCATATTATATCTAAATTGTGATTGTGTTGTCTTAGACTTTGGATATTGTAAAATCGTACATATGGTCTTGTCC
TGATTTTAAAGGCTGCATTACAGCAAGGTGATATAATTTTCTGAACTGACCAGACTGTTCTCGCATTTTTATTA
TCTGACTTTACCTACTTAGTCATTATATTCCCATTACTGATGATATTATCAATATTAAGGTATATTGTCAAAAATAT
TTCATTTTTTCCATATTGTCCAGCCCTAGACATGATGGATATGTATCAAAAAGGACAAGTTTTATTTATGTATGT
ATTTTTTAATACTGCATAGAGTTAATCAGAATTTACAGAAGAATGTTGTCATGATCACTTCGCCCTTGAGCCCT
CATTTAGCCAGATTAATTAAGATTATCATACTTGAAGCTGCACTAATCAATACTTTTCTACAAAAAATGGGTAAT
GTAAAATATGTCACTCCTGGTGACAAACACAGACAGTCTGCAGTTCCCCAGAAGCTGAAAGAGCGTTTTAG
CGTCTTTCACCTCATTGTTTTGGTTTTACAGCCCAAAAGTTTTGTCACTCTCATCAGTGTTGCTAGCGGCTGTT
TCCAAACCAAAAAAAGAGCTTTGAAAAACTCACTGTACGCTACCTGCTCAGCTCCACATGATGGACAAAGTT
GGTAAATAGTGCAGTTAGCAGATAAAGAGTCCGATATTCCCATCAAGAGTTGGTGGAGACCAAAACTGGAC
TAAAAGAAGAATGAGTATTTGACTTACTAATGTTGTTCTGTGTTCGCCAGCAGCCCTGTCTCCAGAAATTACA
TTCATGTACAACTAATTTGAAACAGTAGGTGTGCTTTGTGGGAAATGTAAAGCTGAGGGATTTATGTTTTTTA
TTAACATAATGAGGAACCATCTGTCAAGCTGCAAAAATGTTACATTACAGCAAAATGTTCACTGGAAACATAT
TTCAGTTGTGTTCAGCCATAATAAGCAATCACGATCTTTCCCTAACCTTAACCAAAGTGCATTTGTTGCCTAAA
CCTAAGACATGAGACAGCATCTGAACCCAAAGTTCTAGTGTCAAGGTCGTGCACTTTGTACACCTGCAGTGT
CCCCCAAACACCTCTTTACTAAGTTTATGCAAAAGATGAATGCAGTGTTTCAATACCTGACAAAAAATGTGTC

TAACAACATAATTCAGGCAGTGGCTACATTGCCACAACATAATTAAGAAAGCAGGTTAGTAACATACAGATGT
CATTTATAGGAGACAGAGTTGCTGCTGGATGTATAAATAGGCAAGTGTTTGCTAATATTTTCACCATATCATCT
TGAAAAGGTGCACAATATGTCAGTCATGTGTGCAAGGTTAATTTTTTTGTAGTTTCCCACCTTGAAATGGCCA
GAGGGAAAAAACAGTAGTCATGCTGCTTGAAATTATTGTTCTTTCTGTTTCCAGGTGACAA

>grouper srebp-1 ORF

ATGAATAGCCTGTCTTTTGACGATCCCTCGTTGGATAACCTGGATCCAACGCTGTCGCTTAATGACCCCAGCG
ATATTGACACGGCCCTCTTAAGCGACATTGATGACATGCTACAGCTCATCAGCAACCAGGACATGGAGTTTG
GAGGACTGTTTGATAACCCTCCATACACAGCACCTCCTCCCAGCCAAGAGCATCCTGGTCTGACTCAGTCCAT
CACCTCATCTGCCCCTCCAACCGCCACTACCACACCTCCAACTCCACCCTCATCTTCCTCTTCCATCCTAAGCA
GTAGCCCCCACCTGGATGCGCTCCTGGGCCCTCCCATCACCCGTAGCTCATCCACCCCGGACAAGGCCTTCC
AGCCTCCCACCTTCCAGCAATCCCCCCTGGCCCAGGTGCCCAGCTCCACGCAGAGGCAGCAGCCGTCATCC
CCACAGCAGGCTCAGAGCCTCAGACAGCCCCAGGTGGAGCAGCCCCAACCCATTCTCAGCCCGCCTGCCAC
GGCTCAGGCAGCGTCACCTCATGGCTCACCAGCACCAAACCCAGCTTTCAGCGCCACACCCCAGGCTCTCT
TCACCTCGCCTACTCCCCAGACTCCACCTCAGCCTCAGCCACAGCCGCAGACACAGCTTCAGGCTCAGCCCC
AACAGGTCCGGACCAACTACAGCAACCAGAACGGCTACACAGCTGGCAGTCCCAGCACTGTGAGCCAACC
CACCACCATCCTGTCATCATCACCTCCAACTGTTCAGCCAGTCACCATCCAGGCTCAGCTCCAAGGGCTGACC
ACCACTACTCCTCTCCTGGCCACGTCGGCAAGCCCGCCGGTTCAAACCATCGCACCACACGTACAGCAAGTA
CCTGTGTTGCTGCAGCCCCAGTTCATCAAGGCTGATTCTCTGCTGCTGACCACTCTGAAGCACGACCCCTGT
ATTGTCACGACTATGGCCTCTCCCACATCGCTGGCCACCACCACCCCAGTACAGAGCACTTCACTGCAGGCT
TTTATGGGCAGTGGAACCATCCTGACCACGGTGCCCGTCATGGTGGACACTGAGAAGCTGCCCATCAACCG
CATCGCTATCACCGGCAAGCCAGCAGGCCAGCCGCACAAGGGCGAGAAGCGCACGGCTCACAATGCCATC
GAGAAGCGCTACCGCTCCTCTATTAATGATAAGATCATTGAGCTCAAAGATCTGGTGGCTGGCACTGAGGCC
AAGCTCAACAAGTCTGCAGTGCTGAGGAAAGCCATCGACTACATCCGTTACCTGCAGCAGTCCAACCAGAA
ACTCAAGCAGGAGAACATGGCTTATAAAATGGCAGCCCAGAAAAACAAGTCTCTCAAGGACCTGGTTGCCA
TGGAGGTGGACGGACCAGCTGATGTGAAGAACGAGCTGCCCACCCCGCCAGCCTCTGACGTGGGCTCCCC
CACCTCTTTCTCGCACTGTGGCAGTGACTCAGAGCCTGACAGTCCCATGGGGGAAGACACTAAGCCGAGCG
TGGGCGTGTTAGACAGATCAGCAGCAGGAGGCAGCGCTGGCGGTATGTTGGACCGTTCCCGCATGGCGTT
GTGCGCCTTCACCTTCCTCTTCCTTTCCCTCAACCCGCTGGCTGCCCTGCTCTGCTCATCCAGCAGCAGCTCG
GCTGGAAGCGCAGCCGCCACTGCCACCCATCATGCAGGAAGAACCATGCTGGGTGTGGAGATCGCAGCGG
ACTCGTGGGGCTGGATGGACTGGATGCTGCCGACTATACTGGTGTGGCTGCTCAACGGTATTCTGGTGTCAG
GGGTTCTGATCCGACTGTTAGTGTACGGAGAGCCTGTCACCAGACCACACTCTGGATCGTCTGTCTTGTTCT
GGAGGCACCGCAAGCAGGCTGACCTGGACCTCGCTAGAGGAGATTTCGCCCAGGCCAGTCAGAACCTGTG
GACCTGTCTAAAGGCTCTTGGTCGTCCCTTACCCATCTCCCAGTTGGACCTGGCATGCGCCGCACTCTGGTCC
CTGCTAAGATTCTGTCTGCAGCGCCTCTGGGTGGGCCGCTGGCTGGCTGCCAGGGCCGGAGGGCTACGATC
TGACCGCCCCCTGAAGGAAGACGCCTGTAAAAGCAGCCGGGATGCCGCTCTGGTTTACCACCGCCTGCACC
AGCTTCACATGACAGGTAAGCTGAATGGTAGCCACCTGTCAGCGGTGCACATGGCTCTGAGTGCAGTGAAC
CTGGCAGAGTGCGCTGGCTCCTGTCTGCCTGTAGCCAGTCTGGCTGAGGTCTACGTCTCAGCAGCTCTACGG
GTCAAAGCCAGCCTGCCAAGGATCCTGCATTTTACCTCACGTGTGTTCCTGAGCAGCGCCCGCCAGGCGTG
CCTGTCATCCAGTGGCAGTGTGCCTCCAGCTATGCAGTGGTTGTGTCACCCACTCGGTCACCGCTTCTTTGTG
GATGGGGACTGGGCTATTCGCAGCACTCCTAAAGAAAGCATCTACAGCCAGGCTGGCAATACTGTGGATCC
TCTGGCTCAGGTGACTCAGGCGTTCAGGGAACACCTCCTGGAGAAAGCTCTGTACTGTGTTGCCCAGCCAC
ACGAAGGGAAAAGCTCAAACCAGGGCGAGGGGGAGTATGCTGATGCCCTGGAGTACCTCCAGCTGTTGAT
CAGTGCTTCAGATGCGGCTGGTGCCACCTCCCAGTCTTTTGCTATTGGCTCCAACATGGCCACTGTGACTGG
CTGTGACCCCCACTCCAAGTGGTGGTCCTCAGTTGCCGTGGTGATCATCAACTGGCTCCAAGGAGACGACA

CCGCGGCAGAGAGACTGTATCCAACTGTTGAGCACCTGCCCCGCAGCCTGCAGAACGCAGAGAGTCTTCTG
CCCAAGGCATGTCTGAACACATTCAGAGCGGTGCGGGCGCTGCTGTCCAAGCCGGAAAACTGCCAGCTGA
GTCTGAGCTACAGCGACAAGGCCAACGCCCTGCTTCGAGACAGCCTCAACCTAGGACCACACTGCCACAGC
TCCAGTTTAGACAAGGTTATCCAGTTGCTCTTGTGCGATCTTCTGTTGGTGATGAGGACCAATGTGTGGCGC
CTGCAGCAGCAGGGGGCGGGTCCTGCAGGGTCAGGGACGACTGGTACCAGCGGCCCTGCGGGGGTCCAC
CAGGCCTCCCCACCGGAGCTCCAAGGCTTTCAGCAAGATCTCAGCTCCCTACGCAAGCTGGCACACAGCTT
CAGGCCTGCAATGCGGAGGTTGTTCCTTCATGAAGCTACAGCCAGGCTGATGGCGGGGGCCAGTCCCACCC
GCACACATCAGCTCCTGGATCGCTCGCTGCGACGCAGGGCAACGCCCGGAGCCAAGACAGAGGAGTGCG
AGACGCGGCCAGGCCAGCGGGAGCAGGCTGAGGCAGTGATGCTGGCGTGCCGCTACCTTCCCCCCTCCTT
TCTGTCGGCTCCCGGCCAAAGGGTGGGCATGCTGGCGGACGCGGCCCGCACCCTGGAGAAGCTGGGAGA
CAAGAGGACCCTCCACGACTGCCAGCAAATGATCATCAAGCTGGGCAGCGGCACTACGGTCACCAACAGCT
AG
