# Supplementary material for: Molecular Cloning, Functional Characterization and Nutritional Regulation of the Putative Elongase Elovl5 in the Orange-Spotted Grouper (Epinephelus coioides)
Source: PLoS One. 2016 Mar 7;11(3):e0150544. doi: 10.1371/journal.pone.0150544 (PMC4780818; doi:10.1371/journal.pone.0150544)
Supplement: S1 Table — (PDF) [file pone.0150544.s002.pdf]

S1 Table : Formulation and proximate analysis of the experimental diets (% dry weight).

Ingredients(%)	Dietary n-3 HUFA contents (% dry weight)				
	0.52	0.94	1.57	1.97	2.43
Casein[1]	13	13	13	13	13
Defatted white fish meal[1]	35	35	35	35	35
Defatted krill meal[1]	10	10	10	10	10
Squid meal[1]	5	5	5	5	5
Hydrolyzed fish meal[1]	8	8	8	8	8
LT-Yeast[1]	2	2	2	2	2
Alginate sodium	2	2	2	2	2
α-starch	5	5	5	5	5
Vitamin premix[2]	1.5	1.5	1.5	1.5	1.5
Mineral premix[3]	1.5	1.5	1.5	1.5	1.5
Attractant[4]	1.5	1.5	1.5	1.5	1.5
Ethoxyquin	0.1	0.1	0.1	0.1	0.1
Choline chloride	0.2	0.2	0.2	0.2	0.2
DHA enriched oil[5]	0.33	1.45	2.56	3.66	4.8
EPA enriched oil[6]	0.02	0.66	1.31	1.95	2.58
Palmitin[7]	9.75	7.99	6.23	4.49	2.72
ARA enrich oil[8]	1	1	1	1	1
Soy lecithin	4	4	4	4	4
Mold inhibitor[9]	0.1	0.1	0.1	0.1	0.1
Proximate analysis (n=3)					
Crude protein (%)	57.98	57.21	57.15	57.20	57.71
Crude lipid (%)	15.09	15.24	15.11	15.46	15.75
Ash (%)	16.08	16.11	15.29	15.30	15.19

[1] Casein: crude protein 87.91% dry matter, crude lipid 1.69% dry matter;Defatted fish meal: crude protein 73.36% dry matter, crude lipid 1.52% dry matter; Defatted Krill meal: crude protein 71.80% dry matter, crude lipid 2.93% dry matter;Squid meal: crude protein 61.72% dry matter, crude lipid 3.16% dry matter;Hydrolyzed fish meal: crude protein 77.10% dry matter, crude lipid 4.60% dry matter.

[2]Vitamin premix(IU or g kg^{-1} vitamin premix): retinal palmitate, 3,000,000 IU; cholecalciferol, 1,200,000 IU; DL–α–tocopherol acetate, 40.0 g; menadione, 8.0 g; thiamin–HCl, 5.0g; riboflavin, 5.0 g; D–calcium pantothenate, 16.0 g; pyridoxine–HCl, 4.0 g; meso–inositol, 200.0 g; D–biotin, 8.0 g; folic acid, 1.5 g; para–aminobenzoic acid, 5.0 g; niacin, 20.0 g; cyanocobalamin, 0.01 g; ascorbyl polyphosphate (contained 25% ascorbic acid), 100.0 g.

[3]Mineral premix（g kg^{-1}）：$Ca(H_2PO_4)_2 \cdot H_2O$, 675.0; $CoSO_4 \cdot 4H_2O$, 0.15; $CuSO_4 \cdot 5H_2O$, 5.0； $FeSO_4 \cdot 7H_2O$, 50.0; KCl, 50.0; KI, 0.1; $MgSO_4.2H_2O$, 101.7； $MnSO_4 \cdot 4H_2O$, 18.0; NaCl, 80.0; $Na_2SeO_3 \cdot H_2O$, 0.05; $ZnSO_4 \cdot 7H_2O$, 20.0.

[4]Attractant(g $100g^{-1}$): betaine, 50; glycine,15; alanine, 10;argine,10; taurine, 10; inosine-5’-monophosphoric acid, 5.

[5]DHA enriched oil: DHA content, 40.64% of TFA; in the form of DHA-methylester; JIANGSU TIANKAI Biotechnology Co., Ltd., China.

[6]EPA enriched oil: EPA content, 46.41% of TFA; DHA content, 23.66% of TFA; both in the form of triglyceride; HEBEI HAIYUAN Health biological Science and Technology Co., Ltd., China.

[7]Palmitin: Palmitic acid content, 98.7% of TFA, in the form of methylester; Shanghai Zhixin Chemical Co., Ltd., China.

[8]ARA enriched oil: ARA content, 53.69% of TFA, in the form ofof ARA-methylester; JIANGSU TIANKAI Biotechnology Co., Ltd., China.

[9]Mold inhibitor: contained 50% calcium propionic acid and 50% fumaric acid.
