# Supplementary material for: Molecular Cloning, Functional Characterization and Nutritional Regulation of the Putative Elongase Elovl5 in the Orange-Spotted Grouper (Epinephelus coioides)
Source: PLoS One. 2016 Mar 7;11(3):e0150544. doi: 10.1371/journal.pone.0150544 (PMC4780818; doi:10.1371/journal.pone.0150544)
Supplement: S2 Table — (PDF) [file pone.0150544.s003.pdf]

S2 Table: Fatty acid composition of the experimental diets (% total fatty acids)

Fatty acid	Dietary n-3 HUFA contents (% dry weight)				
	0.52	0.94	1.57	1.97	2.43
14:0	1.17	1.51	2.08	2.35	2.72
16:0	69.64	59.20	47.90	44.71	34.05
18:0	1.50	1.81	1.98	1.91	2.10
20:0	0.73	0.85	1.05	1.09	1.21
∑SFA[1]	73.04	63.37	53.01	50.06	40.08
18:1	6.23	6.47	6.87	6.63	6.96
∑MUFA[2]	6.23	6.47	6.87	6.63	6.96
18:2n-6	8.36	8.95	10.76	9.00	9.25
18:3n-6	0.38	0.27	0.45	0.34	0.40
20:4n-6	2.81	3.20	3.48	3.41	3.77
∑n-6PUFA[3]	11.55	12.42	14.69	12.75	13.42
18:3n-3	1.01	1.08	1.41	1.02	1.06
18:4n-3	0.29	0.32	0.44	0.37	0.43
20:5n-3	1.48	3.11	5.34	6.71	8.56
22:6n-3	2.92	6.09	11.04	13.78	18.22
∑n-3PUFA[4]	5.70	10.59	18.23	21.88	28.27
n-3/n-6PUFA	0.48	0.85	1.25	1.73	2.14
n-3LC-PUFA[5]	4.40	9.20	16.38	20.49	26.78
DHA/EPA[6]	1.98	1.96	2.07	2.05	2.13
EPA/ARA[7]	0.53	0.97	1.53	1.97	2.27

[1]SFA: saturated fatty acids.

[2]MUFA: mono-unsaturated fatty acids.

[3]n-6 PUFA: n-6 poly-unsaturated fatty acids.

[4] n-3 PUFA: n-3 poly-unsaturated fatty acids.

[5]n-3 LC-PUFA: n-3 highly-unsaturated fatty acids.

[6]DHA/EPA: 22:6n-3/20:5n-3.

[7]EPA/ARA:20:5n-3/20:4n-6
